# Anxiety and Depression Among Patients With Different Types of Vestibular Peripheral Vertigo

**DOI:** 10.1097/MD.0000000000000453

**Published:** 2015-02-06

**Authors:** Qing Yuan, Lisheng Yu, Dongmei Shi, Xingxing Ke, Hua Zhang

**Affiliations:** From the Department of Otolaryngology (QY, HZ), The First Affiliated Hospital of Xinjiang Medical University; Department of Otolaryngology (QY, DS, XK), The People's Hospital of Xinjiang Uygur Autonomous Region, Xinjiang; and Department of Otolaryngology (LY), Peking University People's Hospital, Beijing, China.

## Abstract

Numerous studies have been published on comorbid anxiety and depression in patients with vertigo. However, very few studies have separately described and analyzed anxiety or depression in patients with different types of vestibular peripheral vertigo. The present study investigated anxiety and depression among patients with 4 different types of peripheral vertigo.

A total of 129 patients with 4 types of peripheral vertigo, namely, benign paroxysmal positional vertigo (BPPV, n = 49), migrainous vertigo (MV, n = 37), Menière disease (MD, n = 28), and vestibular neuritis (VN, n = 15), were included in the present study. Otological and neurootological examinations were carefully performed, and self-rating anxiety scale and self-rating depression scale were used to evaluate anxiety and depression.

Patients were divided into 2 groups, according to the vestibular function: normal and abnormal vestibular function. There was no significant difference in the risk of anxiety/depression between these 2 groups. However, for patients with the 4 different vertigo types, the prevalence of anxiety (MV = 45.9%, MD = 50%) and depression (MV = 27%, MD = 28.6%) was significantly higher in the patients with MV or MD than those with BPPV or VN (*P* < 0.05).

Vestibular function is not significantly associated with the risk of anxiety/depression. Anxiety/depression is more common in patients with MV or MD than those with BPPV or VN. This may be due to the different mechanisms involved in these 4 types of vertigo, as well as differences in the prevention and self-control of the patients against the vertigo.

## INTRODUCTION

Vertigo is a common clinical syndrome, and the annual prevalence and incidence is about 5% and 1.4% in adults, respectively. The most common causes of vertigo are peripheral vestibular disorders.^1^ Clinical practice has demonstrated that many patients with vestibular peripheral vertigo also have depression/anxiety. The coexistence of these disorders could lead to a vicious circle and have a serious affect on the treatment efficacy and quality of life.

High rates of coexistence of psychiatric disorders especially depression/anxiety and vestibular disorders have been described since antiquity.^2^ However, very few studies have paid attention to the differences between various organic vertigo syndromes with regard to psychiatric comorbidity. In this study, anxiety and depression were evaluated and analyzed in 4 subgroups (benign paroxysmal positional vertigo [BPPV], migrainous vertigo [MV], Menière disease [MD], and vestibular neuritis [VN]) to investigate if there are different incidences of comorbid anxiety/depression in patients with various vestibular peripheral vertigo syndromes, and analysis the possible reasons for the findings.

## PATIENTS AND METHODS

### Patients

Patients, aged 18 to 65 years, with any of the 4 types of vestibular peripheral vertigo syndromes (BPPV, MV, MD, and VN) who were treated in the outpatient department of vertigo, 2nd Department of Otorhinolaryngology, People's Hospital of Xinjiang Uygur Autonomous Region, Xinjiang, China, between August 2013 and June 2014 were included. General characteristics and a medical history of vertigo of each patient were collected. Carefully otological and neurootological examinations (including positioning test, stepping test, head-impulsive test, and head-shaking test), vestibular function tests (monocular electronystagmography, including spontaneous nystagmus, optokinetic nystagmus, rotational chair test, and temperature test), necessary audiometric testing, and psychological assessment were also performed.

The study protocol was approved by the local ethics committee (20140228-04), and all participants provided written informed consent.

### Exclusion Criteria

Patients with >1 of the following features were excluded: acute or chronic vestibular central disorders or central eye movement disorders (including cerebral infarction, cerebral hemorrhage, and multiple sclerosis) within the past 6 months; use of drugs, including benzodiazepines and barbiturates, that could affect the central nervous system within 1 week before inclusion; a history of psychiatric disorders or currently receiving psychological therapies; acute mental disorders or cognitive impairments induced by central nervous system disease; and any special stressful life events experienced within 6 months before inclusion.

### Diagnostic Criteria of the 4 Types of Peripheral Vertigo

#### BPPV

The patients with defined history of positional vertigo and positive result on Dix–Hallpike test were included.^3^ Patients with history of positional vertigo but without a positive Dix–Hallpike test were not included; patients with other types of central positional vertigo were also excluded.

#### MV

There is no universally accepted diagnostic criteria for MV until members of the International Headache Society in collaboration with members of the Barany Society have published diagnostic criteria for it.^4^ Patients diagnosed with definite MV were included if they met the following criteria: episodic vestibular symptoms of at least 5 episodes of moderate or severe vestibular symptoms including rotational vertigo, motion illusion, positional vertigo, other illusory self, or subject motion; migraine according to the diagnostic criteria of the Headache Classification Committee of the International Headache Society^5^; at least 1 of the following migrainous symptoms during ≥2 vertiginous attacks: migrainous headache, photophobia, phonophobia, visual aura, or other aura; and other causes of vertigo ruled out by appropriate investigations.

#### MD

The patients were diagnosed with definite^6^ Ménière disease made on the basis of at least 2 spontaneous episodes of rotational vertigo lasting at least 20 minutes, audiometric confirmation of a sensorineural hearing loss, along with tinnitus, and/or a perception of aural fullness. Most other vestibular conditions were excluded, but further investigation is also necessary to exclude other pathologies.

#### VN

VN was diagnosed according to previous studies,^7^ and the criteria used in the present study were as follows: typical medical history for VN; neurological signs including spontaneous nystagmus, ipsilateral body tilts, and pathological head-impulsive test results; vestibular function tests that showed reduced response in ipsilateral temperature test; and other causes that were excluded.

### Anxiety and Depression Evaluation

Zung self-rating anxiety scale (SAS) and self-rating depression scale (SDS) were used to evaluate anxiety and depression.^8,9^ The 2 scales both have good reliability and validity among Chinese populations.^10^ The SAS is composed of 20 items with 4 responses: “1” indicates “no or a little of the time,” “2” indicates “some of the time,” “3” indicates “good part of the time,” and “4” indicates “most of the time or all the time.” The raw score was standardized according to the formula: standard score = int(1.25 × raw score). A higher summed score represents more severe anxiety symptoms. The cutoff score was 50, which means that standard scores >50 indicate the presence of anxiety. In detail, a standard score of 50–59 indicates mild anxiety, a standard score of 60–69 indicates moderate anxiety, and a standard score of >70 indicates severe anxiety.

The SDS is composed of 20 items with 4 responses also: “1” indicates “no or a little of the time,” “2” indicates “some of the time,” “3” indicates “good part of the time,” and “4” indicates “most of the time or all the time.” The raw score was standardized according to the formula: standard score = int(1.25 × raw score). A higher summed score represents more severe depression symptoms. The cutoff score of SDS was 53, which means that standard scores >53 indicate depression. In detail, a standard score of 53–62 indicates mild depression, a standard score of 63–72 indicates moderate depression, and a standard score of >72 indicates severe depression.

### Statistical Analysis

SPSS 13.0 software, SPSS Inc, Chicago, IL, was used for the statistical analyses. One-way analysis of variance (ANOVA) was used for the analyses of quantitative data with normal distribution, whereas Mann–Whitney was used for analysis of the data with nonnormal distribution. χ^2^ test was used for the comparison of ≥2 rates; Pearson correlation was used to investigate the associations among different variables. *P* < 0.05 was considered statistically significant.

## RESULTS

A total of 129 patients with peripheral vertigo including 44 males and 85 females were included, the mean age of the patients was 47.08 ± 9.901 years; 49 patients (17 males and 32 females) had BPPV, 37 patients (9 males and 28 females) had MV, 28 patients (10 males and 18 females) had MD, and 15 patients (9 males and 6 females) had VN (Table 1).

**TABLE 1:**
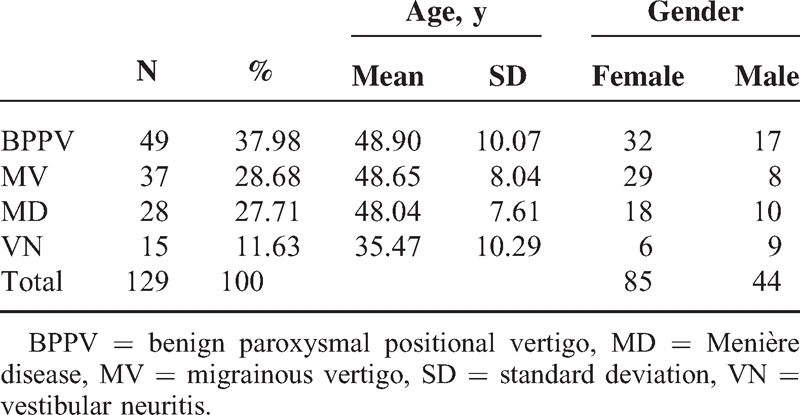
Patient Demographics

### Vestibular Function

Vestibular function of these patients is shown in Table 2. Among the 49 patients with BPPV, 5 (10.2%) had reduced or no response to the ipsilateral temperature test and 1 had vestibular neuritis 8 months prior; no other abnormality was found except in the positioning test. Among the 37 patients with MV, 10 (27.2%) had slight isolated central nystagmus (nystagmus induced by looking upward), oscillating splitting nystagmus, and abnormality in smooth pursuit eye movements; 8 of the 37 patients (21.6%) had abnormalities in temperature test, among which 5 (13.5%) had reduced response in the unilateral temperature test and 3 (8.1%) had reduced response in bilateral temperature tests. No abnormality was found in other tests. Twenty one (75.0%) of 28 patients with MD had obvious reduced or no response in the ipsilateral temperature test, no abnormality was found in other vestibular function tests; 12 (80%) of 15 patients with VN had substantially reduced or no response in the ipsilateral temperature test, and another 3 patients had the response reduced by 17% to 21% in temperature test, all of 15 patients showed spontaneous nystagmus and abnormality in head-shaking test. Patients were divided into normal vestibular function group and abnormal vestibular function group according to their vestibular functions. Mann–Whitney rank-sum test was used and found no significant difference in the rate of anxiety/depression between the 2 groups (*P* = 0.898 and 0.967, respectively).

**TABLE 2:**
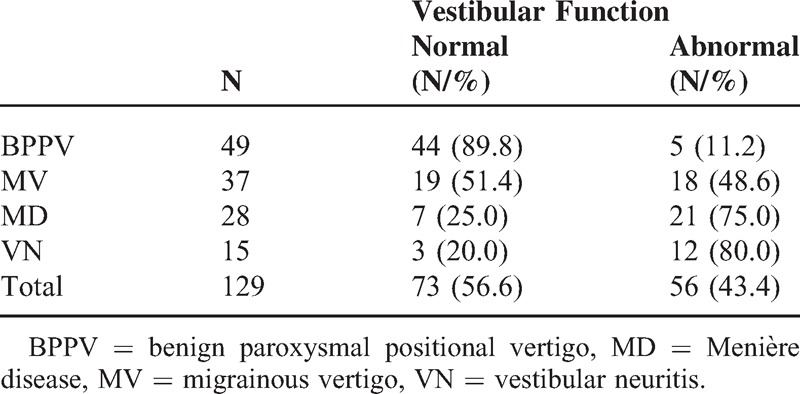
Vestibular Function

### SAS and SDS Scores

SAS scores of patients with MV and MD were significantly higher than patients with BPPV and VN. One-way ANOVA showed that the difference among the 4 groups was statistically significant (F = 3.048, *P* = 0.031); further post hoc analysis showed a significant difference between BPPV and MV (*P* = 0.011) and between BPPV and MD (*P* = 0.023) (Tables 3 and 4).

**TABLE 3:**
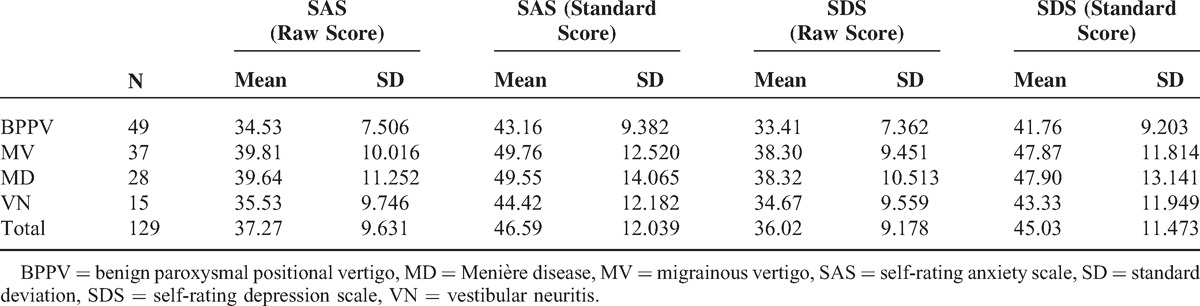
SAS and SDS Scores and Comparison

**TABLE 4:**
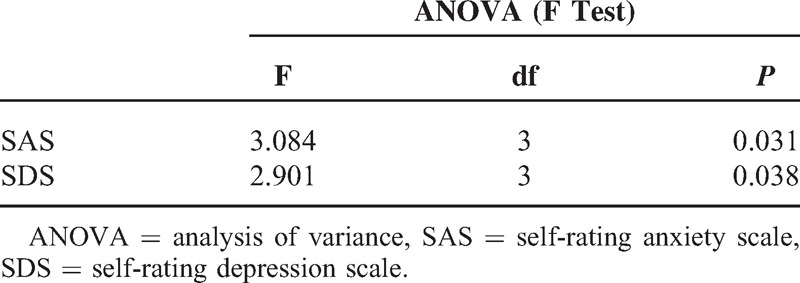
Comparison of SAS and SDS

SDS scores of patients with MV and MD were significantly higher than patients with BPPV and VN. One-way ANOVA showed that the difference among the 4 groups was statistically significant (F = 2.901, *P* = 0.038); further post hoc analysis showed a significant difference between BPPV and MV (*P* = 0.014) and between BPPV and MD (*P* = 0.023) groups (Tables 3 and 4).

### Comparison of Anxiety/Depression

**FIGURE 1:**
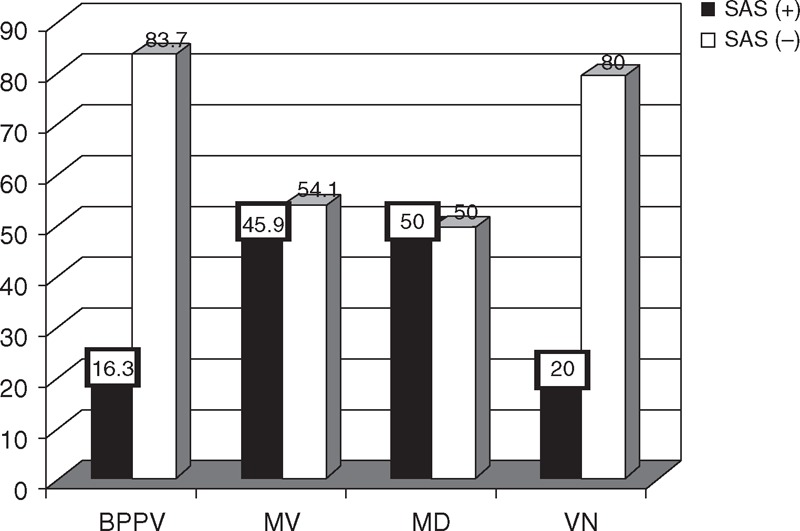
Comparison of anxiety among the 4 groups. BPPV = benign paroxysmal positional vertigo, MD = Menière disease, MV = migrainous vertigo, VN = vestibular neuritis.

**FIGURE 2:**
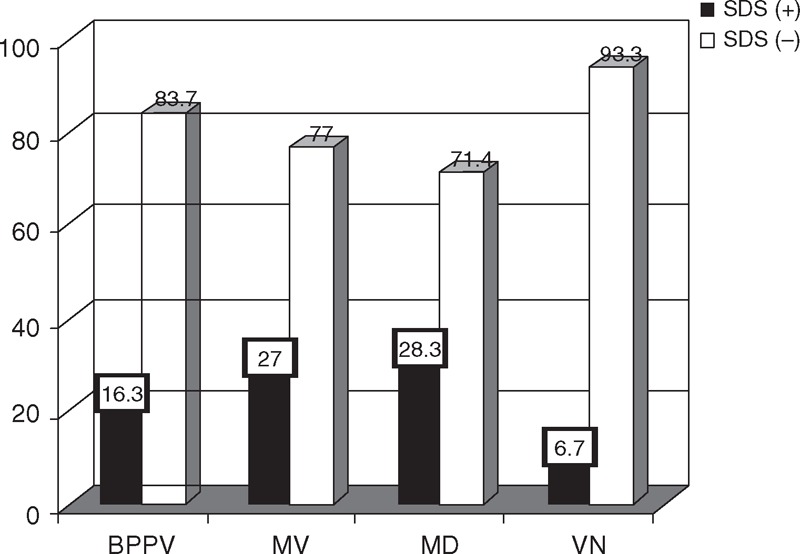
Comparison of depression among the 4 groups. BPPV = benign paroxysmal positional vertigo, MD = Menière disease, MV = migrainous vertigo, VN = vestibular neuritis.

The percentage of both anxiety and depression in MV and MD were significantly higher than in BPPV and VN (Table 5, Figures 1 and 2), and the difference in the risk of anxiety and depression was statistically significant among the 4 groups (*P* = 0.01 and 0.04, respectively). Paired comparisons showed that the differences between BPPV and MV groups, BPPV and MD groups, and MD and VN groups were statistically significant (*P* < 0.05).

**TABLE 5:**
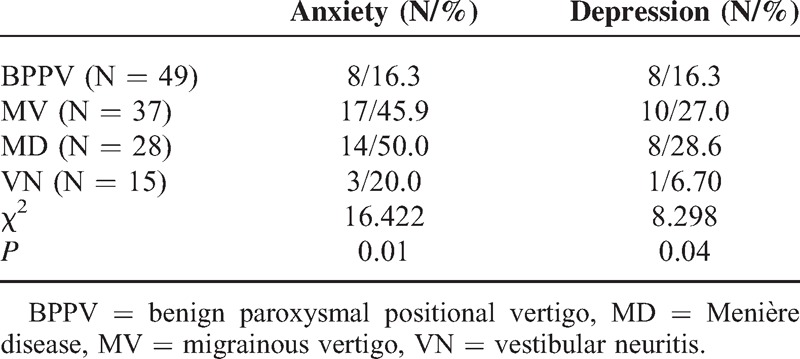
Comparison of Anxiety/Depression Among the 4 Groups

## DISCUSSION

To date, no universally accepted epidemiological data are available to describe the incidence of each type of vestibular peripheral vertigo. The most commonly accepted classification criteria for vestibular peripheral vertigo are BPPV, MV, MD, and VN, as described in the present study. Previous studies have demonstrated that about 80% of patients’ daily life were severely affected by recurrent vertigo.^1^ Emotional disorders especially anxiety and depression are also frequently reported in patients with organic vertigo syndromes,^11^ which could, in turn, aggravate vertigo.

In order to exclude the effects of age-related disequilibrium and emotional disorder, the patients included in the present study were restricted between 18 and 65 years of age. The distribution of ages and genders of the patients were similar to other studies. The percentage of males was slightly higher in the VN group, whereas for the other 3 groups, more females were included. The mean age of the patients in the VN group was significantly lower than in the other 3 groups, of which the mean ages were approximately 48 years. Previous studies have suggested that vestibular dysfunction could be an important cause of emotional disorders, including anxiety and depression.^12–14^ However, Best et al^15^ performed a 1-year perspective study in 68 patients with acute vestibular vertigo and found no association between the severity of vestibular dysfunction and the occurrence and severity of anxiety and depression, which was in accordance with the findings of Liu et al.^16^ In the present study, we also found no association between the vestibular function and the occurrence of anxiety or depression in patients with vertigo; therefore, we suggest that the severity of vestibular dysfunction should not be used as a evaluation indicator for the occurrence of anxiety and depression in vertigo patients. The variable conclusions from different studies could be because of the different interpretation of vestibular parameters. Slow spontaneous nystagmus occurs in about 20% healthy individuals, and it is considered to be pathological, only when the speed of spontaneous nystagmus reaches 5 to 6°/s.^17^ Reduced reactions in the unilateral temperature test may suggest central vestibular compensation after the previous vestibular dysfunction. Therefore, we could not diagnose vestibular disease simply based on the difference or obvious dominance between the left and right sides in vestibular temperature tests, positive results in the rotatory test, or separate spontaneous nystagmus. Instead, vestibular vertigo could be diagnosed only when pathological spontaneous nystagmus, gaze-evoked nystagmus, reduced response in the temperature test, medical records, and the results of neurootological examination are considered as a whole. If any single abnormality in vestibular function tests is considered to be defective, the chance of controversial conclusion among different studies would be greatly increased.

Although no significant difference in the prevalence of anxiety or depression was found between the individuals with normal and abnormal vestibular function, the prevalence of anxiety or depression was significantly different among the patients with BPPV, MV, MD, and VN.

There are a number of possible reasons for the differences between the subgroups. Previous studies have demonstrated the connections between vestibular nerves and several motion-related regions including parabrachial nucleus (PBN), locus coeruleus (LC), dorsal raphe nucleus, and central nucleus of infralimbic cortex, and PBN could connect with motion-control regions including the central nucleus of amygdala, infralimbic cortex, and hypothalamus furthermore.^18^ In addition, connections between vestibular nuclei and the hippocampus, frontal lobe, and dentate gyrus have also been reported.^19^ Abnormal vestibular stimulations could result in increased release of several neurotransmitters that play important roles in anxiety and depression including serotonin (5-HT), dopamine (DA), and norepinephrine (NA) through the connection with PBN, LC, and DRN.^18,20^ Although BPPV, MV, and MD are all recurrent episodic vertigo diseases, the mechanisms of vertigo involved are different. The mechanism involved in BPPV is mechanical stimulation to the semicircular canal ampulla induced by the detached otoconia from the utricle. Histologic studies have given evidence for endolymphatic hydrops as the direct cause of MD. During the attack, the perilymph becomes contaminated with potassium-rich endolymph due to the rupture or leakage of the distended endolymphatic membrane, resulting in an intoxication of vestibule–cochlear hair cells. The mechanism of MV is still debated, probably central and peripheral factors all come into play, it has been postulated that central nervous system dysfunction induced abnormal activity of trigeminal caudate nucleus, solitary nucleus, and vestibular nuclei,^21^ as well as asymmetric release of neurotransmitters including 5-HT, NA, and DA increased by abnormal trigeminovascular pathways,^22^ and calcium channel disorders in brain and inner ear might all be responsible for symptoms of headache and vertigo. Therefore, these different mechanisms for different diseases could be the major cause of the differences in the incidence of anxiety/depression in BPPV, MV, and MD.

We postulate the reason for the higher incidence of anxiety/depression could be that the abnormal vestibular signal stimulating the motion-related regions to release more neurotransmitters intermittently in MV and MD is not only in the attack stage but also in the intermission.

Although anxiety and depression in the VN, MV, and MD groups are induced by the release of neurotransmitters in the above-mentioned connections, the prevalence of anxiety/depression was significantly lower in the VN group than in the MV and MD groups. We propose that some time is needed for the accumulation of these neurotransmitters, and anxiety/depression could be evoked only when the accumulation of neurotransmitters through continuous abnormal stimulation exceeded a certain threshold. The attack of vertigo in VN patients is mainly acute and sustained for about 1 week; recurrent vertigo is rare. Vestibular compensation is more easily established in central vestibular structures, which could rebalance the neural activity and restrict the abnormal stimulation of emotion-regulatory regions. The disease duration are typically >6 months for the patients with MD or MV, and the episodes of acute vertigo are not continuous but paroxysmal that means the imbalance in the afferent activity is paroxysmal and fluctuating; thus, vestibular compensation cannot be easily established. Without vestibular compensation, the rebalance between bilateral neural activities difficult to be established, the continuous abnormal stimulation of the emotion-regulator regions would persistently exist, which could be another reason for the different prevalence of anxiety and depression between the VN and MV or MD groups.

We also believe that the self-control of the patients against the vertigo is associated with the occurrence of anxiety/depression. BPPV patients could control the severity and even the attack of vertigo by avoiding the quick head shaking; vestibular adaptation could develop with the elongation of the disease course, which could alleviate the symptoms of vertigo in BPPV patients. However, for patients with MV or MD, vertigo is unpredictable and uncontrollable, which makes it hard to avoid. In addition, inappropriate treatment could increase the frequency and severity of vertigo and thus make patients more nervous and frightened, and much concerned about vertigo. In our experience, every episode of unpredictable vertigo could induce a panic-like disorder substantially adding to the psychological burden of patients, and, in turn, increase the risk of anxiety/depression. Compared with the patients with BPPV or VN, patients with MD or MV always show many concomitant symptoms such as migraine, tinnitus, hearing loss, and recruitment or hyperacusis in addition to vertigo. As a cause of anxiety/depression, migraine and anxiety/depression share common genetic and environmental risk factors and the interaction between migraine and anxiety/depression has been found.^23^ The auditory center and limbic system are comprehensively associated with motion-control regions too. Persistent tinnitus, fluctuating hearing loss, and recruitment or hyperacusis could increase the psychological burden and thus also evoke anxiety/depression.^24^ Auditory system is anatomically and physiologically connected with the vestibular system, so hearing disorders accompanied with vertigo could also be a reason for the increased incidence of anxiety/depression in patients with MV or MD.

## CONCLUSION

The findings of the present study suggested that comprehensive analyses of the results of vestibular function tests and neurootological examinations are very important for diagnosing peripheral vertigo. However, vestibular function could not be used as the index to evaluate anxiety and depression in these patients. Compared with VN and BPPV, anxiety and depression was more frequent in patients with MV and MD. Therefore, we recommend that clinicians need to be aware of the increased risk, and therefore the potential need to treat psychological disorders where they are identified and diagnosed. Scales should be used to screen for anxiety/depression in these patients to aid in the early diagnosis and treatment, and psychologists/psychiatrist should be considered to help with the diagnosis and treatment, if necessary. Cognitive-behavioral therapy and rehabilitation of vestibular function or drug therapy are also necessary for the improvement of quality of life. Further differentiated investigations are required to show the possible neuroanatomical and neurobiological factors involved in the higher risk of anxiety and depression, as well as the higher frequency of anxiety in patients with MV and MD.
